# Increased percentage of T cells with the expression of CD127 and CD132 in hypertrophic adenoid in children with otitis media with effusion

**DOI:** 10.1007/s00405-012-1977-8

**Published:** 2012-03-02

**Authors:** Beata Żelazowska-Rutkowska, Jolanta Wysocka, Karol Ratomski, Edwina Kasprzycka, Bożena Skotnicka

**Affiliations:** 1 Department of Pediatric Laboratory Diagnostics, Medical University of Bialystok, Waszyngtona 17, 15-274 Bialystok, Poland; 2 Department of Pediatric Otolaryngology, Medical University of Bialystok, Waszyngtona 17, 15-274 Bialystok, Poland

**Keywords:** Adenoid, IL-7R, Lymphocytes T and B

## Abstract

The hypertrophic adenoid may promote chronic suppurative otitis media in children as it fulfills its immune function. The number of lymphocytes in the adenoid and their cooperation in the immune response depend of on their proliferation and migration to the effector sites. Interleukin 7 (IL-7) is essential for the normal development and function lymphocytes. IL-7 plays pivotal role for activation and proliferation of T and B cells. The heterodimeric interleukin-7 receptor (IL-7R) is composed of the IL-7Rα (127) and the common cytokine receptor γc (CD132). The aim of this study was to evaluate the percentage of lymphocytes T (CD4^+^ and CD8^+^) with IL-7R (CD127 and CD132) expression in hypertrophic adenoid in children suffering with otitis media with effusion for a duration of 3 months. Adenoid excised due to hypertrophy with or without chronic otitis media with effusion was used as study material. CD4^+^ CD127^+^, CD4^+^132^+^, CD8^+^CD127^+^ and CD8^+^CD132^+^ cell subpopulations were identified using monoclonal antibodies and flow cytometry. The percentage of CD4^+^ and CD8^+^ T cells with CD127 receptor expression in hypertrophic adenoid of children with otitis media with effusion was statistically significantly higher than in hypertrophic adenoid group. The percentage of CD4^+^ T cells with CD132 expression in the study group was statistically significantly higher than in the reference group. The percentage of CD8^+^ T cells with CD132^+^ expression was not statistically different in both groups. The increased percentage of T lymphocytes with IL-7R expression (CD127 and CD132) in hypertrophic adenoid seems to influence the quantity of lymphocytes and upset the immunological function of tonsils which can influence the course of otitis media with effusion.

## Introduction

Interleukin 7 (IL-7) plays a basic role in the development of lymphocytes in the thymus, in the production of memory T cells and in the homeostasis of peripheral T cells [1–3]. IL-7 is generated by nonhematopoietic stromal cells of many organs, including the thymus, bone marrow and peripheral lymphatic organs [2, 3].

The receptor for IL-7 (IL-7R) is a heterodimer composed of α chain (IL-7Rα or CD127) and the accompanying γ chain (γc or CD132), which is also a receptor for IL-2, IL-4, IL-9, IL-15 and IL-21 [1, 2, 4–6]. The expression of IL-7R (CD127 and CD132) can be found in naive CD4^+^/CD8^+^ T cells and CD4^+^/CD8^+^ memory T cells [7–10]. IL-7 binding to the receptor results in the initiation of at least three activation cascades: JAK/STAT (Janus kinase/signal transducer and activator of transcription), PI3 K (Phosphoinositide 3-kinase) and MAPK (mitogen-activated protein kinase)/ERK (extracellular signal-related kinase) [5, 11, 12].

The signal transmitted through the IL-7Rα induces increased expression of apoptotic proteins, mainly Bcl-2 [10, 12–14], cell proliferation [13, 15], antigen-independent naive T-cell proliferation, strong expansion of memory T cells (effector) in the presence and lack of antigen [7, 8, 16]. The IL-7Rα also plays an important role in signal transmission required for the development of the secondary lymphoid system [17]. At the same time it stimulates Fas antigen expression, by increasing T cell sensitivity to apoptosis [18].

The regulation of IL-7Rα expression is of major importance for the efficient production of CD4^+^ and CD8^+^ memory cells, as a result of the immune response. The basic level of IL-7 is sufficient for the maintenance of viability of CD8^+^ memory cells and to some extent contributes to the maintenance of viability of the CD4^+^ memory T cell pool [2].

The study objective was to assess the percentage of lymphocytes T (CD4^+^ and CD8^+^) with IL-7R (CD127 and CD132) expression in the group of children with hypertrophic adenoid in children with otitis media with effusion who suffer of 3 months’ duration.

## Materials and methods

The study material contained adenoid excised from 35 children (18 girls and 17 boys, aged from 3 to 17) suffering from adenoid hypertrophy coexisting with otitis media with effusion (OME) lasting at least 3 months. The reference group consisted of 30 children (15 girls and 15 boys, aged from 3 to 17) with adenoid hypertrophy (HA), but without otitis media, lasting at least 3 months. All children were clinically free of infection at the time of surgery. Due the treatment failure, the children were qualified for adenoidectomy in the Department of Children’s Otolaryngology Medical University of Bialystok. The study was approved by the Bioethical Committee, Medical University of Bialystok (number R-I -002/264/2009).

## Methods

Immediately after excision, the adenoids were placed in RPMI 1640 culture medium containing 10% fetal calf serum (Immuniq). Then, they underwent mechanical grinding so as to give a homogenous suspension. The suspension was centrifuged for 1 min. at 4°C (at 100–150×g). The obtained supernatant containing leukocytes was subjected to further analysis. Cells were obtained from the supernatant through centrifugation for 8 min at 4°C (at 300×g), and then rinsed with PBS containing 0.1 mM EDTA and 0.02% NaN_3_ (Sigma) and centrifuged again for 8 min at 4°C (at 300×g).

The density of cell suspension and cell morphology was assessed both under a microscope and using a hematologic analyzer Sysmex XT 2000i. Cell suspension of 4–10 × 10^3^/μl density was used for analysis. The isolated mononuclear cells in the form of suspension in PBS were incubated with monoclonal antibodies conjugated with fluorochromes directed against antigens (CD4-PE-Cy5, CD8-FITC, CD4-FITC/CD8PE/CD3-PerCP, CD127-PE, CD132-PE) (Beckman Coulter). The samples were evaluated in a flow cytometer Coulter PC500, equipped with argon laser emitting at 488 nm.

### Statistical analysis

Non-parametric U Mann–Whitney test was used for statistical analysis. The differences for *p* < 0.05 were considered statistically significant. The results were described as the mean, standard deviation, median, minimum and maximum values and lower *P*_25_ and upper *P*_75_ quartiles.

## Results

**Fig. 1:**
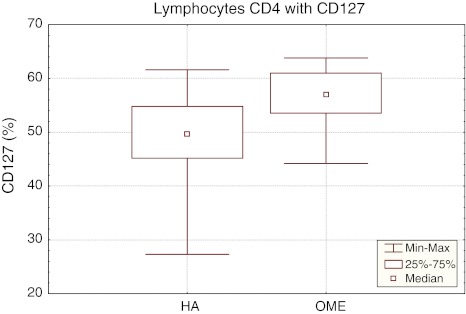
The percentage of CD4^+^CD127^+^ lymphocytes in the reference (HA) and study (OME) group. Median, result range, *P*_25_ i *P*_75_

The mean percentage of CD4^+^ T cells with CD127 expression in hypertrophic adenoid with otitis media with effusion (OME) was 56.37 ± 5.73%, being statistically significantly higher (*p* < 0.005) than in the reference group (HA 48.18 ± 9.41%) (Table 1). In OME group, the median of the percentage of CD4^+^CD127^+^ lymphocytes was 57.05%, *P*_25_ 53.60% and *P*_75_ 61.00%, with result range of 44.20–63.80%. The confidence interval for the percentage of CD4^+^CD127^+^ lymphocytes in the reference group (HA) ranged from 45.20% (*P*_25_) to 54.80% (*P*_75_), median 49.65% and result range of 27.30–67.60% (Fig. 1).

**Table 1:** Percentages of lymphocytes CD4^+^CD8^+^ with expression CD127^+^ and CD 132^+^ receptor in hypertrophic adenoid in children with and without otitis media with effusion

Subpopulation of lymphocytes X ± SD (%)	G Reference group (HA) *N* = 18	St Study group (OME) *N* = 16
CD4^+^ CD127^+^	48.18^A^ ± 9.41	56.37^B^ ± 5.73
CD8^+^ CD127^+^	48.72^C^ ± 9.03	56.53^D^ ± 11.87
CD4^+^ CD132^+^	39.91^E^ ± 7.54	45.44^F^ ± 8.13
CD8^+^ CD132^+^	44.60 ± 9.21	50.48 ± 11.75

A vs B *p* < 0.005

C vs D *p* < 0.04

E vs F *p* < 0.05

**Fig. 2:**
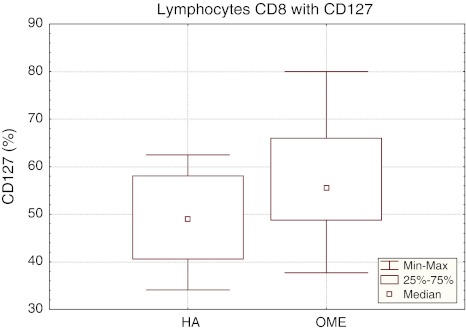
The percentage of CD8^+^CD127^+^ lymphocytes in the reference (HA) and study (OME) group. Median, result range, *P*_25_ i *P*_75_

The mean percentage of CD8^+^ lymphocytes with CD127 expression in OME children was 56.53 ± 11.87%, being statistically significantly higher (*p* < 0.04) than in reference group (HA 48.72 ± 9.03%) (Table 1). In the study group, the median of the percentage of CD8^+^CD127^+^ lymphocytes was 55.6%, with *P*_25_ 48.80% and *P*_75_ 65.90% and the range of results 37.70–80.00%. The confidence interval for the percentage of CD8^+^CD127^+^ lymphocytes in the reference group (HA) ranged from 40.60% (*P*_25_) to 62.50% (*P*_75_) with the median equal to 49.00% and the range of results from 34.10 to 67.60% (Fig. 2).

**Fig. 3:**
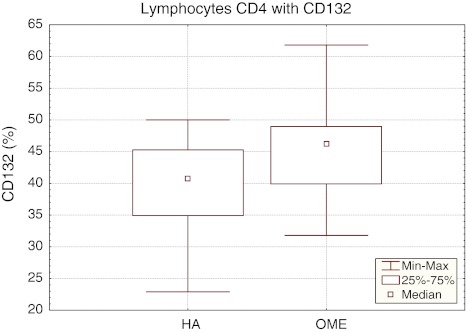
The percentage of CD4^+^CD132^+^ lymphocytes in the reference (HA) and study (OME) group. Median, result range, *P*_25_ i *P*_75_

The mean percentage of CD4^+^ lymphocytes with CD132 expression in hypertrophic adenoid in children with otitis media with effusion was 45.44 ± 8.13%, being statistically significantly higher (*p* < 0.05) than in the reference group (HA 39.91 ± 7.54%). In the study group (OME), the median of the percentage of CD4^+^CD132^+^ cells was 46.20%, *P*_25_ 39.90% and *P*_75_ 49.00%, with the range of results from 31.80 to 61.80%. The confidence interval for the percentage of CD4^+^CD132^+^ lymphocytes in the reference group (HA) ranged between 39.40% (*P*_25_) and 45.30% (*P*_75_), median 40.80% and the range of results from 22.90 to 50.00% (Fig. 3).

**Fig. 4:**
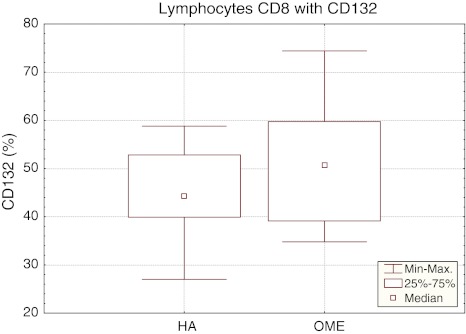
The percentage of CD8^+^CD132^+^ lymphocytes in the reference (HA) and study (OME) group. Median, result range, *P*_25_ i *P*_75_

The mean percentage of CD8^+^ T cells with CD132 expression in OME children was 50.48 ± 11.75%, being higher than in the reference group (44.6 ± 9.21%). The difference was not statistically significant (Table 1). In the study group (OME), the median of the percentage of CD8^+^CD132^+^ cells was 50.75%, *P*_25_ 39.10%, *P*_75_ 59.80% and the range of results from 34.80 to 74.40%. The confidence interval for the percentage of CD8^+^CD132^+^ cells in the reference group ranged from 39.90% (*P*_25_) to 62.50% (*P*_75_), median 44.25% and the range of results between 27.00 and 58.80% (Fig. 4).

## Discussion

The interfollicular space of the adenoid is dominated by T cells, both naive and memory lymphocytes, which get there through high endothelial venule (HEV) from peripheral blood. Naive T cells, after penetrating the interfollicular space of the adenoid, get into contact with antigen-presenting cells (APC), which eventually leads to their reactivation, retention in the interfollicular space and proliferation [19]. Some of the naive T cells are converted into effector cells which undergo rapid elimination after pathogen destruction, whereas others into long-lived memory cells [20].

Factors that affect T cell viability vary depending on the state of these cells [21]. One of the major factors is pleiotropic IL-7, produced by stromal cells of many organs [2, 4], as well as by dendritic cells in the peripheral lymphatic organs [22]. Fry et al. [4] claim that since IL-7 is produced by dendritic cells of the peripheral lymphatic organs and T cell response to IL-7 is IL-7R expression dependent, a reduction in the number of lymphocytes does not cause their dysfunction [23].

The percentage of CD4^+^ and CD8^+^ T cells with CD127 receptor expression in hypertrophic adenoid in children with otitis media with effusion was statistically significantly higher than in HA group. The percentage of CD4^+^ T cells with CD132 expression in the study group was statistically significantly higher than in the reference group. However, the percentage of CD8^+^CD132^+^ T cells in the study group was only slightly higher compared with the reference group.

IL-7 is known to regulate T cell viability. When bound to its receptor (CD127) on CD8^+^ lymphocytes it increases the expression of anti-apoptotic proteins and induces the conversion of effector cells into long-lived memory cells [10]. On the other hand, Fluur et al. [18] have shown that IL-7 also stimulates Fas expression and its level correlates with T cell sensitivity to apoptosis. Previously, we have demonstrated that lymphocyte apoptosis in hypertrophic adenoid in children with otitis media with effusion more enhanced than in the HA group, which seems to be due to the fact that in hypertrophic adenoid of OME children the percentage of CD4^+^ and CD8^+^ lymphocytes with Fas expression was higher compared with the group without the inflammation, whereas the percentage of these cells with Bcl-2 expression was lower [3].

As IL-7 takes part in the regeneration of T cells, their increased sensitivity to this cytokine is essential for the maintenance of homeostasis. Previously, we have found that hypertrophic adenoid of children with otitis media with effusion exhibits markedly lower percentages of CD4^+^ and CD8^+^ T cells [24]. Therefore, a high percentage of T cells with IL-7R expression in children with otitis media with effusion may contribute to the maintenance of normal proportions in the subpopulations of adenoid T cells. At the same time it may lead to the formation of a considerable number of memory cells.

## Conclusion

The increased percentage of T lymphocytes with IL-7R expression (CD127 and CD132) in hypertrophic adenoid seems to influence the quantity of lymphocytes and upset the immunological function of tonsils which can influence the course of otitis media with effusion.
